# Ketogenic diet therapy in developmental and epileptic encephalopathies: toward syndrome- and gene-informed precision therapy

**DOI:** 10.3389/fped.2026.1958733

**Published:** 2026-09-23

**Authors:** Debopam Samanta, Christopher Paul Manbeck

**Affiliations:** Department of Pediatrics, Division of Child Neurology, University of Arkansas for Medical Sciences, Little Rock, AR, United States

**Keywords:** developmental and epileptic encephalopathy, drug-resistant epilepsy, infantile epileptic spasms syndrome, ketogenic diet, precision medicine

## Abstract

**Background:**

Developmental and epileptic encephalopathies (DEEs) are severe, early-onset, frequently drug-resistant epilepsies in which recurrent seizures and epileptiform activity contribute to cognitive impairment beyond the underlying etiology. Ketogenic dietary therapies (KDTs) exert broad, multimodal antiseizure and neuroprotective effects and occupy a distinct position among nonpharmacologic treatments for DEEs.

**Objective:**

To synthesize the evidence for KDT across the principal electroclinical DEE syndromes, an expanding spectrum of gene-specific DEEs, and acute presentations such as febrile infection-related epilepsy syndrome (FIRES) and new-onset refractory status epilepticus (NORSE), with attention to outcome measures, mechanisms, adverse effects, and monitoring.

**Methods:**

A narrative review of the literature on the classic ketogenic diet (KD), modified Atkins diet, medium-chain triglyceride diet, and low-glycemic-index treatment in DEEs, drawing on randomized controlled trials, cohort studies, systematic reviews, and meta-analyses.

**Results:**

KDT has the strongest and most methodologically rigorous evidence base in infantile epileptic spasms syndrome, including comparative-effectiveness data vs. hormonal therapy, followed by strong consensus supporting early use in epilepsy with myoclonic-atonic seizures. Meaningful benefit has also been reported in Dravet syndrome and, to a somewhat lesser extent, Lennox–Gastaut syndrome. Responses are more variable in early-infantile developmental and epileptic encephalopathy, epilepsy of infancy with migrating focal seizures, developmental and epileptic encephalopathy with spike-wave activation in sleep, and hemiconvulsion–hemiplegia–epilepsy syndrome, with tolerability and long-term adherence often declining over time. In FIRES/NORSE, KDT may serve as an acute anti-inflammatory treatment for super-refractory status epilepticus. Gene-specific responses vary substantially, with the most favorable outcomes observed in transporter-related disorders, followed by conditions involving other membrane-associated proteins, including ion channels and neurotransmitter receptors, whereas disorders affecting cellular structural integrity or homeostatic regulation appear less responsive.

**Conclusions:**

KDT has an increasingly well-supported, syndrome- and endpoint-specific role across DEEs. Future research should prioritize comparative-effectiveness trials, standardized outcome measures, predictors of response, better-tolerated formulations, and equitable access to dietary therapy.

## 1 Introduction

Developmental and epileptic encephalopathies (DEEs) comprise a heterogeneous group of severe, early-onset epilepsies in which frequent epileptiform activity and recurrent seizures contribute to cognitive impairment beyond the developmental consequences of the underlying etiology (1). The 2017 and 2022 International League Against Epilepsy classification frameworks distinguished developmental encephalopathy, epileptic encephalopathy, and combined developmental and epileptic encephalopathy, while recognizing the increasingly identifiable genetic etiologies of these disorders (2, 3). Syndromes within this spectrum—including early-infantile DEE, epilepsy of infancy with migrating focal seizures, infantile epileptic spasms syndrome (IESS), Dravet syndrome, epilepsy with myoclonic-atonic seizures, Lennox–Gastaut syndrome, DEE with spike-wave activation in sleep, hemiconvulsion–hemiplegia–epilepsy syndrome, and febrile infection-related epilepsy syndrome (FIRES)—are frequently pharmacoresistant, with many affected children continuing to experience seizures despite two or more appropriately selected antiseizure medications (ASMs). For these drug-resistant epilepsies, dietary therapies—particularly ketogenic dietary therapies (KDTs)—have demonstrated additional benefit beyond standard ASMs in clinical trials and therefore occupy a distinct position in the therapeutic landscape of DEEs (4–6). Unlike most ASMs, which primarily target specific ion channels or neurotransmitter systems, KDTs exert broad, multimodal effects (Table 1). This mechanistic breadth may help explain their efficacy across etiologically diverse DEEs.

**Table 1:** Proposed mechanisms of action of KDT in DEEs.

Mechanistic domain	Proposed mechanism	Relevance to DEE
Alternative cerebral fuel supply	Ketone bodies (beta-hydroxybutyrate, acetoacetate) cross the blood-brain barrier independently of GLUT1 and are metabolized to acetyl-CoA, bypassing impaired glucose transport or pyruvate oxidation	Disease-specific mechanism in GLUT1 deficiency syndrome and pyruvate dehydrogenase complex deficiency; broader relevance to any state of relative cerebral energy insufficiency
Enhanced mitochondrial bioenergetics	Increased mitochondrial biogenesis, upregulated tricarboxylic acid cycle flux, and improved ATP-to-ADP ratio, raising the seizure threshold by stabilizing neuronal membrane potential	Broadly applicable across DEE syndromes; may be particularly relevant in mitochondrial and metabolic DEEs
Modulation of neuronal excitability	Ketone bodies and reduced glycolytic flux enhance opening of ATP-sensitive potassium (K-ATP) channels and shift excitatory/inhibitory balance toward inhibition. ketogenic therapy likely increases adenosine availability and A_1_-receptor–mediated inhibition	Proposed contributor to broad-spectrum antiseizure effect across DEEs, including channelopathies
GABAergic/glutamatergic modulation	Ketosis increases glutamate decarboxylase activity and GABA synthesis while limiting glutamate-mediated excitotoxicity	Particularly relevant to syndromes with prominent generalized and myoclonic seizures (EMAtS/Doose, SLC6A1-DEE)
mTOR pathway inhibition	KD suppresses mammalian target of rapamycin (mTOR) signaling, converging with mTOR-inhibitor pharmacotherapy	Specific relevance in tuberous sclerosis complex-associated DEE
Anti-inflammatory/innate immune modulation	Beta-hydroxybutyrate inhibits NLRP3 inflammasome activation, reducing IL-1β and TNF-α production and attenuating innate immune signaling	Central to the rationale for KD in FIRES/NORSE and other presumed inflammatory/immune-mediated encephalopathies
Reduced oxidative stress	Ketone metabolism increases NADPH availability and glutathione synthesis, reducing reactive oxygen species and neuronal injury	Proposed neuroprotective contribution across DEEs, potentially relevant to preservation of developmental trajectory
Gut microbiome modulation	KD alters gut microbial composition in ways that have been linked to seizure protection in preclinical models	Emerging area; mechanistic contribution to human DEE outcomes not yet well characterized

The rationale for the present review is threefold. First, the past decade has generated substantial new evidence regarding KDTs in DEEs, including randomized controlled trials comparing KDT with hormonal therapy in IESS, large multicenter cohorts in Dravet and Lennox–Gastaut syndromes, and an expanding literature on KDT as an acute intervention in FIRES. Second, the increasing identification of gene-specific DEEs through clinical genomic sequencing has raised important questions regarding whether response to KDT varies according to molecular etiology and whether KDT should be introduced earlier for selected monogenic DEEs within a precision-therapy framework (7, 8). Third, existing reviews of KDT in pediatric epilepsy often either consider DEEs as a homogeneous group or focus narrowly on individual syndromes, leaving clinicians without a unified resource that compares syndrome-specific efficacy, relevant outcome measures, mechanistic rationale, adverse-effect profiles, and monitoring requirements.

This narrative review, with its methodology summarized in Table 2, consolidates the available evidence for the principal KDT approaches (Table 3)—including the classic ketogenic diet (KD), modified Atkins diet (MAD), medium-chain triglyceride (MCT) diet, and low-glycemic-index treatment (LGIT)—across major electroclinical DEE syndromes and an expanding spectrum of gene-specific DEEs, with particular attention to the outcome measures most relevant to each condition.

**Table 2:** Summary of the narrative review search strategy.

Component	Detail
Databases searched	PubMed/MEDLINE, Embase, Cochrane CENTRAL; hand-search of reference lists and recent journal issues
Date range	Inception to July 2026, with emphasis on studies published 2015–2026
Core search terms	“ketogenic diet” OR “modified Atkins diet” OR “medium-chain triglyceride diet” OR “low glycemic index treatment” AND syndrome/gene-specific terms
Syndrome terms	Early-infantile DEE/Ohtahara, EIMFS, infantile spasms/West syndrome, Dravet syndrome, myoclonic-atonic epilepsy/Doose syndrome, Lennox-Gastaut syndrome, DEE-SWAS/ESES/Landau-Kleffner, hemiconvulsion-hemiplegia-epilepsy syndrome, FIRES/NORSE/super-refractory status epilepticus
Gene terms	SLC2A1/GLUT1, PDHA1, TSC1/TSC2, CDKL5, KCNT1, STXBP1, KCNQ2, SCN2A, SCN8A, PCDH19, SLC6A1, CHD2, SYNGAP1, HNRNPU, SHANK3, UBE3A, MECP2, PLPBP/ALDH7A1
Study designs included	Randomized controlled trials; prospective and retrospective cohorts; multicenter registries; systematic reviews/meta-analyses; case series/case reports (selectively, for ultra-rare syndromes)
Exclusion criteria	Non-DEE populations; dietary effect not separable from concurrent ASM change; syndrome-specific outcome data not extractable
Synthesis approach	Narrative synthesis organized by syndrome/gene; no independent quantitative pooling; existing meta-analytic pooled estimates reported as published

**Table 3:** Overview of ketogenic dietary therapy (KDT) formulations used in DEEs.

Ketogenic dietary therapy	Macronutrient framework	Typical initiation and supervision	Monitoring and supplementation	Patients/settings in which it may be useful	Principal strengths	Important limitations
Classic ketogenic diet	Approximately 90% of energy from fat, with about 5% each from protein and carbohydrate	Usually initiated with close dietitian involvement; inpatient initiation remains common	Baseline contraindication screening, ongoing laboratory monitoring, and micronutrient supplementation are required	Particularly suitable for tube-fed children and for disorders with established responsiveness, including Glut1 deficiency syndrome, pyruvate dehydrogenase complex deficiency, Dravet syndrome, Angelman syndrome, epilepsy with myoclonic-atonic seizures, infantile epileptic spasms syndrome, tuberous sclerosis complex, FIRES, mitochondrial complex I deficiency, and super-refractory status epilepticus	Can be delivered as formula, purée, or solid food and has the broadest clinical experience	Highly restrictive; food portions generally require precise weighing
MCT ketogenic diet	Approximately 30–70% of energy from fat, including 30%–60% from medium-chain triglycerides; protein and carbohydrate allowances are higher than with the classic diet	Dietitian supervision is required, but outpatient initiation is feasible	Baseline and follow-up laboratory assessment and supplementation are generally required	May be considered when the classic diet is poorly tolerated or when a larger food volume is desirable	More liberal and often more palatable than the classic ketogenic diet	Requires incorporation of MCT oil into meals and snacks; higher MCT exposure can cause gastrointestinal adverse effects
Modified Atkins diet	Protein is unrestricted; calorie counting is generally unnecessary; carbohydrate intake is usually limited to 10–20 g/day	Commonly started as an outpatient; dietitian input is advisable but not always mandatory	Laboratory screening and follow-up are recommended, although less intensive than with the classic diet; routine micronutrient supplementation is not always necessary	Often practical for toddlers, school-aged children, adolescents, and adults who need greater dietary flexibility; may also be easier to implement in resource-limited settings	Lower caregiver burden, simpler outpatient initiation, and greater flexibility than traditional ketogenic diets	Carbohydrate restriction remains substantial, particularly early in treatment; the apparent flexibility may contribute to dietary lapses, and fat intake still remains relatively high
Low glycaemic index treatment	Protein and calories are not routinely restricted; carbohydrate intake is generally limited to 40–60 g/day, with foods selected to maintain a glycaemic index below 50	Usually suitable for outpatient initiation and may be implemented without intensive dietitian involvement	Routine laboratory surveillance and supplementation are less consistently required	May be useful in adolescents with dyslipidaemia, adults with multisystem comorbidities, patients at risk for renal stones, and vegetarian or vegan households; may also be more feasible in low-resource settings	Offers the greatest flexibility and lowest implementation burden	Requires careful identification of low-glycaemic-index foods, may produce limited ketosis, and is not appropriate when sustained ketosis is essential, such as in Glut1 deficiency syndrome or pyruvate dehydrogenase complex deficiency

FIRES, febrile infection-related epilepsy syndrome; GLUT1 deficiency syndrome, glucose transporter type 1 deficiency syndrome; MCT, medium-chain triglyceride.

Absolute contraindications to ketogenic dietary therapies: primary carnitine deficiency; carnitine palmitoyltransferase I or II deficiency; carnitine-acylcarnitine translocase deficiency; fatty-acid β-oxidation disorders, including medium-, long-, and short-chain acyl-CoA dehydrogenase deficiencies and medium- or long-chain 3-hydroxyacyl-CoA dehydrogenase deficiencies; porphyria; and pyruvate carboxylase deficiency.

## 2 Ketogenic diet therapy across core electroclinical DEE syndromes

The following sections review the evidence for KDT across the principal electroclinical DEE syndromes, organized by syndrome.

### 2.1 Early-infantile developmental and epileptic encephalopathy (EIDEE)

Early-infantile developmental and epileptic encephalopathy (EIDEE), encompassing disorders previously classified as Ohtahara syndrome and early myoclonic encephalopathy, is among the earliest-onset and most severe DEEs. It typically presents during the first three months of life with tonic or myoclonic seizures and a burst-suppression EEG pattern.

Evidence supporting KDT in EIDEE is limited primarily to small case series and individual case reports, reflecting both the rarity of the disorder and the practical challenges of implementing dietary therapy during the neonatal and early-infantile periods. Nevertheless, KD has produced clinically meaningful responses in selected patients, particularly in association with specific genetic and metabolic etiologies.

Reported responsive conditions include *ALG13*-, *SCN2A*-, and *KCNQ2*-related epilepsies; mitochondrial respiratory-chain disorders; *SLC6A8*-related creatine transporter deficiency; and glycine encephalopathy/nonketotic hyperglycinemia (9–18). In some reports, seizure improvement was accompanied by variable functional benefits, including increased alertness and improvements in feeding or muscle tone. In one series, 53.8% of patients with *STXBP1*-related EIDEE experienced a reduction in seizure frequency, while 27.1% achieved seizure freedom following KDT (18). In the same study, five patients with *SCN2A*- or *KCNQ2*-related disease had reduced seizure frequency, including three patients with *KCNQ2* variants who became seizure-free. A separate case report described complete resolution of both seizures and EEG abnormalities in an infant with *SCN2A*-related EIDEE after KD was initiated at 39 days of age (9). Another patient with EIDEE of unknown etiology experienced a marked and sustained reduction in seizures and emergency department visits for approximately one year, although the burst-suppression EEG pattern persisted (13).

Interpretation of these findings remains difficult. Genetic etiologies such as *STXBP1*, *ALG13*, and *SCN2A* can produce several distinct electroclinical phenotypes, including IESS, and syndrome-specific outcomes are not always clearly delineated within published case series (19, 20). In addition, spontaneous seizure reduction or remission may occur in some genetic forms of EIDEE. When KD is introduced later in the disease course, distinguishing a true treatment effect from the underlying natural history may therefore be challenging.

### 2.2 Epilepsy of infancy with migrating focal seizures (EIMFS)

Epilepsy of infancy with migrating focal seizures (EIMFS) is characterized by frequent or near-continuous multifocal seizures that migrate between cortical regions on EEG, with onset typically before 12 months of age. Gain-of-function variants in *KCNT1* are among the most frequently identified genetic causes (21). Evidence supporting KDT in EIMFS is derived mainly from small case series, individual reports, and caregiver-reported outcomes.

Caraballo et al. reported a favorable response in two of three patients treated with KD (22). One patient achieved seizure freedom accompanied by substantial neuropsychological improvement and no further hospitalizations, while another experienced a 75%–99% reduction in seizure frequency with moderate cognitive improvement (22). Similarly, Armeno et al. described two infants with *SCN2A*-related EIMFS who responded to early KDT: one became seizure-free, and the other achieved a 75%–99% reduction in seizure frequency, with neurocognitive improvement reported in both cases (23). Another study comparing multiple therapies in 50 individuals with *KCNT1*-related epilepsy identified KDT as the most effective treatment (24). In a caregiver survey of 62 families affected by *KCNT1*-related disorders, KD was perceived as one of the most beneficial therapeutic interventions (25). A systematic review identified approximately 70 patients with *KCNT1*-related EIMFS who had received KDT (26). Among the 40 patients for whom outcomes were available, 25 (62.5%) showed some degree of benefit, although responses were frequently described qualitatively rather than using standardized seizure-reduction thresholds (27–33). Despite these potentially favorable findings, treatment was generally initiated relatively late, at approximately 1 year of age on average, and the mean duration of therapy was also approximately 1 year, which limits conclusions regarding the benefits of earlier intervention (26).

Because EIMFS is frequently complicated by status epilepticus and recurrent hospitalization, parenteral ketogenic nutrition has occasionally been used when enteral feeding is temporarily not feasible because of intestinal failure, ileus, impaired gastrointestinal absorption or motility, or feeding-related respiratory compromise. Armeno et al. described short-term parenteral ketogenic therapy in which ketogenic ratios above 1.5:1 could not be achieved because of limitations in fat administration and the need to maintain adequate protein intake (34). Nevertheless, this ratio was sufficient to preserve seizure control while permitting temporary bowel rest. Lipid administration requires careful monitoring and adjustment because of the potential for hepatic and metabolic toxicity.

### 2.3 Infantile epileptic spasms syndrome (IESS)

Infantile epileptic spasms syndrome (IESS; formerly West syndrome) is among the most extensively studied DEE indications for KDT, with evidence derived from retrospective and prospective cohorts, randomized controlled trials, and three independent systematic reviews and meta-analyses (35–71) (Table 4). Adrenocorticotropic hormone (ACTH), vigabatrin (VGB), and oral corticosteroids remain the standard first-line therapies for IESS; however, their adverse-effect burden and the high risk of relapse have sustained interest in alternative approaches, including KDT (72, 73).

**Table 4:** Summary of key studies of KDT in infantile epileptic spasms syndrome (IESS).

Author (year)	N	Study type	Key findings
Dou et al. (35)	56 (KD & MAD arms)	Retrospective case-control (China)	Comparable spasm-reduction rates between MAD and KD at 3, 6, 12 months; ∼75% (KD)/67% (MAD) achieved ≥50% reduction at 3 mo and spasm free 34% (KD) vs. 33% (MAD); MAD had better tolerability/compliance
Dressler et al. (36)	59	Retrospective	67% patients >50% spasm reduction at 3 months
Dressler et al. (37)	101	Randomized controlled trial + parallel cohort	Electroclinical remission at 28 days similar for KD vs. ACTH (RCT: 62% vs. 69%; parallel cohort: 41% vs. 38%); relapse significantly lower with KD in parallel cohort (0% vs. 50%); adverse events requiring intervention markedly lower with KD (30% vs. 94%, including one ACTH-related death)
Dressler et al. (38)	67	KDT vs. ACTH as 2nd treatment	KDT as the second treatment achieved sustained seizure freedom in 50% (12/24), compared to 19% (3/16) with ACTH and 9% (2/22) with ASMs.
Eun et al. (39)	43	Retrospective case series	Seizure-free state in 53.5% (23/43) of patients and a greater than 90% reduction of seizure frequency in 62.8% (27/43) of patients. The seizure outcomes were highly concordant with improvements in EEG findings and development
Freeman et al. (40)	13	Prospective cohort	First prospective KD study including IESS; at 6 months, 8% seizure-free, 31% >90% reduction, 23% 50%–90% reduction, 39% <50% reduction
Hirano et al. (41)	6	Case series (Japan)	KD improved ACTH-resistant West syndrome; disappearance of ES-2/6, >80% reduction-3/6, disappearance of hypsarrhythmia- 5/6
Hong et al. (42)	104	Prospective single-center case series (US)	> 50% spasm improvement occurred in 64% at 6 months and 77% after 1–2 years. Thirty-eight (37%) became spasm-free for at least a 6-month period within a median 2.4 months of starting the KD. In addition, 62% reported improvement in development, 35% had EEG improvement, and 29% were able to reduce concurrent anticonvulsants. Older age at onset of IS and fewer prior anticonvulsants were more likely to be associated with > 90% spasm improvement at 6 months.
Hussain et al. (43)	22	Retrospective case series, highly refractory cohort (US)	Diet initiation beginning a median of 26.4 (12.5–38.7) months after onset, and following a median of 7 (IQR 5–7) treatment failures. Only 2 patients exhibited a complete response during ketogenic diet exposure, and response was more reasonably attributed to alternative therapies in both cases. A modest early reduction in seizure frequency was not sustained beyond 1 month of diet exposure.
Mahesan et al. (44)	87	Randomized controlled trial	Comparable spasm reduction at 6 weeks (KD 76.6% vs. ACTH 66.7%); comparable electroclinical remission (40.5% vs. 36.6%); significantly lower relapse (0% vs. 38.9%) and fewer side effects (59.5% vs. 95.1%) with KD
Meena et al. (45)	80	Randomized open-label trial (MAD vs. topiramate for spasms refractory to steroid and vigabatrin)	MAD significantly superior to topiramate at 12 weeks for >50% reduction (67.5% vs. 42.5%), >90% reduction (22.5% vs. 4.76%), alertness (17.5% vs. 2.5%) and BASED score improvement (40% vs. 12.5%)
Prezioso et al. (46)	341 (13 studies)	Systematic review/meta-analysis	At 6 months, 57.4% had >50% spasm reduction (66% in prospective-study subset); 34% spasm-free overall (29% in prospective subset); 24% seizure-free at 12 months in the 4 studies with long-term data; unknown etiology predicted response
Kang et al. (48)	14 (5 with IESS)	Case series (Korea; mitochondrial complex defects)	KD used safely in mitochondrial disease; 7 became seizure free
Kang et al. (49)	40 (35 evaluable)	Seizure-free outcomes and showed improvement in hypsarrhythmic patterns within 6 months of the KD underwent RCT (short-2 months vs. long-term-2 years KD tapering duration)	Short term- 2/16 relapse and long term- 2/19 progressed to LGS and 1/19-focal seizure
Kayyali et al. (50)	20	Retrospective case series (US)	70% ≥50% reduction at 3 mo; 0% seizure-free at 3 mo, maintained in 6–12 mo. >90% reduction- 20–35%. EEG improvement-60%. Quality of life improvement- 82–100% over 3–12 months.
Kossoff et al. (51)	23	Retrospective case series (Johns Hopkins, US)	At 3, 6, 9, and 12 months, 38%, 39%, 53%, and 46%, respectively, of all patients currently on the diet were >90% improved (3 were seizure-free at 12 months); 67%, 72%, 93%, and 100% were >50% improved. Fifty-six percent remained on the diet at 12 months, 46% of whom were >90% improved and 100% were >50%. Fifty percent of those with hypsarrhythmia and follow-up EEGs had EEG improvement. Fifty-seven percent had their medications reduced or discontinued by 12 months. Fifty-seven percent had improvement in development, which was correlated with seizure control. Independent factors that predicted improvement included age younger than 1 year and previous exposure to 3 or fewer anticonvulsants.
Kossoff et al. (52)	33	Case-control, KD vs. ACTH (new-onset spasms)	Spasm-free in 8 of 13 (62%) in KD vs. 18 of 20 (90%) in ACTH within 1 month; Those treated with ACTH were more likely to have a normal EEG at 1 month (53% vs. 9%, *p* = 0.02), however, use of the KD led to EEG normalization within 2–5 months in all eight who became spasm-free.
Lee et al. (53)	28 (9 spasms)	Case series (Korea; liquid ketogenic milk)	2/9 complete seizure freedom and 8/9 with >50% reduction
Liao et al. (54)	177	Retrospective case series, resource-limited region (China)	54, 47, 27 and 16% ≥50% reduction; 17, 17, 14, and 10% seizure-free at at 3, 6, 12, and 24 mo; long-term neurodevelopmental delays 121/176.
Nordli et al. (55)	17	Retrospective case series (US)	12/17% ≥50% reduction and 6/17 spasm free; early experience using KD in infants(infantile spasms responded better than other seizure types)
Numis et al. (56)	26	Retrospective case series (US)	At 1–3 months after the initiation of the KD, 46% of twenty-six patients had a greater than 90% reduction in IS. No significant relationships between reduction in IS and serum β-hydroxybutyrate, or glucose levels were identified
Pires et al. (57)	17	Prospective case series (France)	After 1 month with KD, 6/17 (35%) patients were seizure free while 11/17 (65%) were seizure-free after the third month.
Rubenstein et al. (58)	13 (infantile spasms 7)	Case series (US)	3 spasms free; 2 >90% reduction
Sanchez et al. (59)	13	Case series (Aicardi syndrome)	Ten (67%) patients experienced a ≥50% seizure reduction after 3 months, with 3 (20%) having a ≥90% reduction. Therapy was more successful in those without spasms.
Sharma et al. (60)	15	Case series, MAD (India)	After three months of diet, six children were spasm free. The time to spasm freedom after diet initiation ranged from two days to two months.
Sharma et al. (61)	91 (46 MAD, 45 control)	RCT, MAD vs. no-diet control (India)	MAD: 11/46 (24%) seizure-free, 30 (62.5%) % ≥50% reduction at 1 month. 9/46 had resolution of hypsarrythmia.
Sharma et al. (62)	40 (20 MAD, 20 KD)	RCT, MAD vs. KD (India)	Similar effectiveness of MAD and KD; spasm freedom 20% vs. 15% at 12 weeks
Ville et al. (63)	23	Case series (France)	4/23 (17%) ≥50% reduction(all 4 had cognitive improvement); KD used successfully combined with corticosteroids
Wu et al. (64)	98	Case series (China)	The spasm-free rate at 3, 6, 12 months of KD treatment was 19.4% (19/98 cases), 20.4% (20/98 cases), 30.6% (30/98 cases). The 3-month spasm-free rate was higher in newly diagnosed patients than patients with prior ASM failure.
Wu et al. (65)	52 (38 infantile spasms)	Prospective case series (China)	11/38 spasm free at 4, 12, and 24 weeks; >50% reduction 20–22/38 over this time period
Yakemow et al. (66)	1,077 (22 studies)	Systematic review/meta-analysis	50%–99% spasm reduction in 48% at 3 months and 41% at 6 months; spasm freedom 25% at both 3 and 6 months; meta-regression did not identify predictors of response
Ye et al. (67)	481	Multicenter retrospective cohort (China)	In different time from initiation (1, 3, 6, and 12 months), the rates of seizure freedom after KD were 6.9, 11.6, 16.0 and 16.8%, respectively. The rates of effectiveness were 44.7, 62.8, 49.1 and 32.0%. The retention rates were 94.0, 82.5, 55.7 and 33.1%. he KD effect was not related to the number of ASMs used before KD startup or prior glucocorticoid failure.
Zhang et al. (68)	135	Multicentre prospective controlled study, N-RCT (China)	The primary outcome was the reduction in spasms and remission of hypsarrhythmia at the 16(th) week, divided into Grade I (spasm-free for at least one week with hypsarrhythmia remission), Grade II (≥50% spasm reduction and/or hypsarrhythmia remission) and Grade III (<50% spasm reduction with hypsarrhythmia). The efficacy in the KD group was superior to that in the control group (Grade I: 13.4% vs. 10.9%; Grade II: 40.7% vs. 20.7%, *p* = 0.025). Patients with a ketogenic ratio <3:1 had a higher rate of Grade I than those with ketogenic ratio ≥3:1 (66.7% vs. 33.3%, *p* = 0.037). No significant correlation was found between the efficacy of KD and level of serum ketosis, aetiology of IS, or age
Zhang et al. (69)	39	Case series (China)	After 3 months of treatment, 20 patients (51.3%) had ≥50% seizure reduction, including 4 patients (10.3%) who became seizure-free. After 6 months of treatment, 4 patients remained seizure free, 12 (30.8%) had 90–99% seizure reduction, 8 (20.5%) had a reduction of 50–89%, and 15 (38.5%) had <50% reduction. Hypsarrhythmia scores were significantly decreased at 3 months of KD. They were associated with seizure outcomes at 6 months independent of gender, the course of disease and etiologies. Patients with a hypsarrhythmia score ≥8 at 3 months of therapy may not be benefited from KD. Maximum total score is 16 for the most severe hypsarrhythmia.
Zhao et al. (70)	74	Single-center cohort	Seizure response 56.8%/41.9%/25.7% at 3/6/12 months; seizure-free rates 13.5%/13.5%/8.1%; retention declined from 70.2% (3 months) to 25.7% (12 months); adverse events in 20.3% (mostly gastrointestinal)
Zuo et al. (71)	1,173 (24 studies)	Systematic review/meta-analysis	≥50% spasm reduction in 63%/64%/60% at 3/6/12 months; complete remission 21%/25%/19%; 54% with hypsarrhythmia/EEG improvement; 56% with neurodevelopmental improvement; 40% experienced adverse effects

Three meta-analyses have synthesized the accumulating evidence. Across analyses pooling 341–1,173 patients from 13 to 24 studies, roughly 55%–65% of children achieved a ≥50% reduction in spasm frequency within the first 3–6 months, with seizure freedom in the 20%–35% range over the same period and modestly attenuated rates by 12 months (46, 66, 71). In the largest of these analyses, more than half of children showed improvement in EEG/hypsarrhythmia (54%) and neurodevelopmental status (56%), though roughly 40% experienced adverse effects. Unknown/cryptogenic etiology predicted a better response in the earliest meta-analysis, whereas the most recent meta-regression did not identify significant predictors of response. Individual studies generally reported comparable response rates, although a subgroup of highly treatment-resistant patients showed little to no sustained benefit (43).

Beyond the ACTH comparisons discussed below, several other randomized designs have addressed specific practical or comparative questions. Kang et al. randomized infants to short (2-month) vs. prolonged (2-year) diet tapering and found low relapse with the brief taper, suggesting shorter courses may be adequate for some responders (49). Among MAD trials, Sharma et al. showed MAD outperformed a no-diet control arm within one month (61), a later head-to-head trial found MAD and KD similarly effective (62), and Meena et al. found MAD superior to topiramate for spasm reduction, alertness, and EEG score improvement in children refractory to steroids and VGB (45).

Two randomized controlled trials have directly compared KD with high-dose ACTH. Dressler et al. found comparable electroclinical remission rates (47% vs. 48%) between KD and ACTH at day 28 in an underpowered study, but a markedly lower relapse rate and substantially fewer adverse events requiring medical intervention with KD (30% vs. 94%, including one ACTH-related death) (37). In infants without prior VGB exposure, electroclinical remission at day 28 was observed significantly less often with KD than ACTH (47% vs. 80%, *P* = 0.02), though time to remission was similar (∼2 weeks) between arms, and age-appropriate psychomotor development at last follow-up favored KD (23% vs. 15%). Among the 67 infants who required a second treatment phase, KD achieved sustained seizure freedom in 50% (12/24), compared with 19% (3/16) for ACTH and 9% (2/22) for ASMs (38). Mahesan et al. similarly found comparable spasm reduction and electroclinical remission between arms, with significantly lower relapse and fewer side effects on KD (44). In ACTH/corticosteroid-resistant IESS specifically, Zhang et al. found KD superior to a control ASM group for spasm/hypsarrhythmia response, with no correlation between etiology and efficacy (68).

Because electroclinical remission—concurrent cessation of spasms and resolution of hypsarrhythmia—is considered a more meaningful endpoint than clinical spasm freedom alone, it is notable that EEG/hypsarrhythmia improvement has consistently lagged behind clinical spasm control by weeks to months, a pattern also reflected in the pooled meta-analytic data above (51, 52). Evidence on neurodevelopmental outcome remains comparatively sparse and inconsistent; the Dressler trial found no difference in long-term neurodevelopmental outcome between KD and ACTH (37).

Several smaller series have examined KD efficacy in specific etiological or clinical subgroups, such as ACTH-resistant IESS, mitochondrial complex defects, trisomy 21, and Aicardi syndrome (41, 48, 59). One study also showed that KD can be safely combined with corticosteroids, although concurrent ACTH or high-dose corticosteroid therapy may be challenging because steroid-induced hyperglycemia increases endogenous insulin, potentially suppressing lipolysis and ketone production (63). Responders also demonstrated accompanying cognitive gains.

Several studies have sought biomarkers or predictive tools for response. Predictors of favorable response identified inconsistently across studies include cryptogenic (unknown) etiology, older age at spasm onset, fewer ASMs trialed prior to KD initiation, and early responsiveness within the first month (35, 39, 42); male sex has been proposed as a predictor in one study but has not been substantiated elsewhere (56). Zhang et al. used a quantitative hypsarrhythmia severity score and found that scores at 3 months predicted 6-month outcome independent of sex, disease duration, or etiology, with scores ≥8 associated with poor benefit—the most direct evidence linking EEG severity to eventual response (69). Numis et al. found no correlation between spasm reduction and serum β-hydroxybutyrate or glucose levels, arguing against a simple dose–response relationship with ketosis depth, while Wu et al. observed higher 3-month spasm-free rates in newly diagnosed children than in those with prior ASM failure, reinforcing earlier-line use as a predictor of benefit (56, 64). However, a larger study of 481 children found no association between KD efficacy and either prior ASM burden or failure of glucocorticoid therapy (67).

Given the tolerability challenges associated with KD, MAD has been increasingly studied as a more liberalized alternative. Across several comparative studies, MAD has demonstrated broadly comparable efficacy to KD for spasm reduction and spasm freedom, generally with improved treatment retention, although direct comparisons remain limited by modest sample sizes (60, 62).

Taken together, the available evidence positions IESS as the DEE syndrome with the strongest and most methodologically rigorous support for KDT, including comparative effectiveness vs. first-line hormonal treatment and meaningful quality-of-life improvements accompanying seizure reduction (50). Among major US pediatric epilepsy centers, 58% specified a defined role for KD in the treatment pathway (74). A scoping review of international IESS guidelines found broad support for KD, with 21 of 28 guidelines (75%) recommending it after first-line treatment failure. Suggested indications included drug-resistant epileptic spasms, inability to use steroids or vigabatrin, and selected metabolic or genetic disorders, such as pyridoxine-dependent epilepsy, pyridoxal−5′-phosphate deficiency, nonketotic hyperglycinemia, *TSC1/TSC2*-related disease, and pathogenic *SCN2A* variants (75).

### 2.4 Dravet syndrome

Dravet syndrome, most commonly caused by pathogenic *SCN1A* variants, is characterized by fever-sensitive and often prolonged convulsive seizures beginning in the first year of life, evolving into a multi-seizure-type drug-resistant epilepsy with progressive developmental impairment (76). Two meta-analyses have specifically examined KD efficacy in this population. Wang et al. pooled seven studies (four retrospective, three prospective) comprising 167 patients, 80%–100% of whom were *SCN1A* pathogenic-variant positive, and found that 63%, 60%, and 47% of responders achieved a ≥50% seizure reduction at months 3, 6, and 12 respectively, with pooled diet retention of 78% at 6 months and 49% at 12 months (77). Li et al. pooled five studies focused specifically on efficacy and adherence, reporting comparable but distinct pooled efficacy of 73.4% at 3 months, 59.0% at 6 months, 44.4% at 12 months, and 51.0% among patients continuing beyond one year, alongside a steep decline in adherence from 90.6% at 3 months to 43.8% at 12 months—suggesting that tolerability, rather than efficacy, becomes the dominant limitation of KDT over time in Dravet syndrome (78).

Among individual cohorts contributing to these pooled estimates, the largest series of 60 patients found the responder rate falling from 62% at 1 month to 28% at 12 months, with the seizure-free rate declining from 28% to 8% over the same period (79). In a multicenter retrospective study of 114 patients, seizure freedom was achieved in 14%–33% of patients during the first year, while ≥50% seizure reduction occurred in 43%–76%. Retention declined from 97.4% at 1 month to 46.5% at 12 months, and adverse events decreased over time (80). In a smaller cohort of 20 children, 13 (65%) experienced a greater than 50% reduction in seizure frequency, with 15 of 20 showing increased alertness and improved cognition and behavior (81).

Among responders, seizure control with KD has consistently been accompanied by parallel improvement in EEG abnormalities (79, 82). Cognitive, developmental, and behavioral benefits have also been reported using various assessment tools, including increases in developmental-age subscores on the Gesell Developmental Schedule (83); improvements in hyperactivity and behavioral disturbances on the Conners and Achenbach scales (84); and parent-reported improvements in cognition, language, and motor function in most treated patients across several cohorts (79, 85). Consistent with these observations, the international consensus on the diagnosis and management of Dravet syndrome identified KD as the therapy most frequently associated with improved alertness or behavior (86). The consensus recommended considering dietary therapy after failure of three or four ASMs and before vagus nerve stimulation: KD was recommended for children aged 6 years or younger with moderate consensus, whereas MAD was strongly recommended for adolescents and adults (86, 87).

### 2.5 Epilepsy with myoclonic-atonic seizures (EMAtS/Doose syndrome)

Epilepsy with myoclonic-atonic seizures (EMAtS, formerly known as Doose syndrome) is characterized by the onset of myoclonic, atonic, and myoclonic-atonic (drop) seizures in previously typically developing children, with a variable but often favorable long-term prognosis when seizures are adequately controlled. Retrospective series report seizure freedom with KD in 18%–58% of patients and a greater than 50% seizure reduction in 35%–60% (88). In the largest study, a French multicenter cohort of 50 children with drug-resistant EMAtS, 54% were seizure-free after 6 months of KD, and 86% had greater than 70% seizure reduction after just 2 months, with earlier diet initiation significantly associated with seizure remission (89). A retrospective analysis of 95 patients similarly found that 79% responded to dietary therapy (KD or MAD), substantially exceeding the 26% response rate to the first three ASMs trialed (90).

Smaller series corroborate this pattern: among 11 patients started on KD after multiple ASM failures, 6 remained on the diet at 18 months, with 2 becoming seizure-free, 2 achieving 75%–99% reduction, and 2 achieving 50%–74% reduction, while the 5 who discontinued did so mainly for lack of efficacy (91). A study using MAD in 30 drug-resistant patients found greater than 50% seizure reduction in 83% and seizure freedom in 47% (92), and another retrospective series found that 7 of 9 patients became seizure-free within weeks of starting dietary therapy (a mix of MAD and KD), with benefit sustained long enough to permit discontinuation of all ASMs (93). Notably, switching from MAD to KD when MAD proves insufficient has been reported to improve seizure control further, with 8 of 9 patients in one series achieving greater than 10% additional seizure reduction and 5 becoming seizure-free (94). Dietary therapy has also been used successfully, with appropriate caution, in a child with EMAtS and coexisting type 1 diabetes, in whom diet-induced and diabetes-related ketosis had to be carefully distinguished (95).

Across this literature, earlier introduction of KD—after failure of three or fewer ASMs—has been associated with significantly improved seizure remission and better cognitive outcomes compared with later introduction, and diet responders are more likely than nonresponders to achieve complete withdrawal of all ASMs (96). Among the DEEs reviewed here, EMAtS carries some of the strongest consensus-level endorsement for early KD use: the international Delphi consensus identifies KDT as an optimal second-line treatment that should be considered early in drug-resistant disease (97).

### 2.6 Lennox-gastaut syndrome (LGS)

Lennox-Gastaut syndrome is characterized by multiple drug-resistant seizure types—tonic, atonic, and atypical absence seizures prominent among them—a slow generalized spike-wave EEG pattern, and cognitive impairment; it is one of the more extensively studied DEE indications for dietary therapy given its high prevalence among drug-resistant childhood epilepsies (98, 99). A meta-analysis of KD in LGS, incorporating 7 articles and 185 patients, found that KD produced meaningful seizure reduction, with 12%, 35%, and 72% of patients achieving seizure freedom, a >75% reduction, and a >50% reduction in seizure frequency, respectively, at 6 months after starting KD (100). A U.S. retrospective cohort of 71 patients found that 51% achieved greater than 50% seizure reduction after 6 months on KD, including 23% with greater than 90% reduction; this effect was sustained at 12 months, and 45% of patients were able to reduce their ASM burden, with generalized seizures appearing to respond particularly well to dietary therapy in this cohort (101). A broader literature review encompassing 18 studies and 189 children with LGS found that 47% achieved a greater than 50% seizure reduction following 3–36 months of KD, and some studies have suggested more favorable responses specifically for generalized tonic-clonic and drop seizures, though this finding is not uniformly reported (101). In a large study evaluating multimodal treatment approaches in LGS, 53 of 201 patients (26.4%) with ASM-resistant LGS achieved seizure freedom for more than 1 year, but only 15 (7.5%) remained seizure-free for more than 5 years with dietary therapy (102). Seizure-free patients also showed improvements in EEG findings and neurodevelopmental status (102).

An Argentinian cohort of 20 children with LGS found that 40% achieved greater than 50% seizure reduction after 18 months of KD, with three patients achieving complete seizure freedom (103). A separate retrospective study of 47 LGS patients reported ∼50% with ≥50% seizure reduction by 3 months, 10% seizure-free and 12.5% with ≥90% reduction by 6 months (104). Long-term follow-up data (mean 19 years) in a cohort of 68 LGS patients found that among 19 who underwent dietary therapy, 26.3% achieved seizure freedom on KD compared with essentially none on MAD (105). Broader DEE cohorts that included LGS subgroups have also reported favorable developmental findings: a prospective study of 65 children with DEEs (including IESS, LGS, EMAtS, and Dravet syndrome) found increases in mean developmental quotient and improvements in attention and social functioning among those remaining on KD at one year, and a separate cohort of 52 children with DEEs, including 7 with LGS, reported cognitive and language improvement in 23 and 7 children respectively (65).

Comparative dietary formulations have also been studied in LGS specifically or in mixed cohorts including LGS. A prospective comparison of KD and MAD in 51 children with refractory epilepsy, 18 of whom had LGS, found no significant overall difference in mean seizure reduction, except in children under 2 years of age, who achieved significantly higher seizure-freedom rates on KD (53% vs. 20%) (106). A retrospective study of 25 children with LGS maintained on MAD found ≥50% seizure reduction in 48% at 3 months, including 2 seizure-free, though over half discontinued by 6 months due to insufficient control or excessive restriction (107). Another prospective study of 14 children with intractable epilepsy including 6 with LGS found that only half continued MAD at 6 months, though 5 of those achieved greater than 50% seizure reduction, including 3 seizure-free; interestingly, higher and more consistent beta-hydroxybutyrate levels (above 3 mmol/L) were associated with better outcomes than fluctuating ketosis in this cohort (47). A larger open-label randomized study of MAD vs. levetiracetam in 101 children, 47 with LGS, found a significantly higher proportion of children with greater than 50% seizure reduction on MAD (52.9% vs. 22%), with numerically but not statistically significantly higher rates of greater than 90% reduction and seizure freedom (6).

Alternative dietary formulations have also been evaluated in mixed refractory-epilepsy cohorts that included substantial LGS subgroups. A randomized trial comparing KD with the MCT diet in 145 children found no significant difference in mean seizure reduction between formulations at any time point up to 12 months, with similar tolerability aside from differences in energy levels at 3 months and vomiting at 12 months favoring MCT and KD, respectively (108). LGIT has shown efficacy in smaller series that included LGS subgroups: an early retrospective study of 20 patients found greater than 90% seizure reduction in half (109), a larger series of 76 children (89% with three or more failed ASMs) found greater than 50% seizure reduction in 66% at 12 months (110), and among a subset of patients with LGS in one Italian series and one Korean series, LGIT produced meaningful responses with response rates comparable to or somewhat lower than reported for KD (111, 112). An open-label randomized trial of LGIT in children aged 2–8 years found 30% with greater than 50% seizure reduction, including one LGS patient achieving seizure freedom, compared with no responders in the control arm (5). In another mixed-epilepsy cohort, patients with LGS had one of the highest responder rates to LGIT, with 8 of 12 patients (75%) showing a favorable response (112). LGIT may be considered for patients who have previously responded favorably to KD, serving as a less restrictive alternative after successful treatment (112, 113). Anecdotal reports suggest that parenteral MCT formulations may be used to maintain ketosis and seizure control in patients with LGS (114).

Consensus recommendations support KDT as an important adjunctive nonpharmacologic treatment for LGS that should be considered early in the course of drug-resistant disease, alongside rational ASM therapy and evaluation for neuromodulation or surgery (115–117). Although seizure-type-specific responses in LGS remain uncertain, response rates may be lower than in some other DEEs, and efficacy for drop seizures may be inferior to corpus callosotomy (118). Nevertheless, its noninvasive and reversible nature, together with potential benefits for neurocognition, alertness, and reduction of polypharmacy, supports its use in appropriately selected patients (119).

### 2.7 DEE with spike-and-wave activation in sleep (DEE-SWAS)

DEE-SWAS, including Landau-Kleffner syndrome, is characterized by marked activation of epileptiform discharges during non-REM sleep, accompanied by cognitive, language, or behavioral regression that is often disproportionate to the overt clinical seizure burden (120). A prospective, single-arm interventional study of MAD in 22 children with confirmed DEE-SWAS (spike-wave index ≥50% during NREM sleep, with seizure or neurodevelopmental regression despite at least two ASMs and corticosteroids) found that seizure remission was achieved in 82%, 90%, and 100% of children remaining on the diet at 4, 12, and 24 weeks, respectively, though EEG response (greater than 50% SWI reduction) was seen in only 33% at 12 weeks, and no significant change in cognitive scores (measured by the Vineland Social Maturity Scale) was observed, despite parent-reported improvements in language (60%) and behavior (40%); compliance was a major limitation, with 54% discontinuing before 12 weeks (121).

This pattern—meaningful seizure reduction with more modest and inconsistent EEG normalization—is broadly consistent with other reports using KD, where seizure reduction rates of 40%–61% have been described, with seizure freedom achieved only in a minority of patients (63) (122). More favorable outcomes, including near-complete or complete seizure freedom and EEG normalization, have been reported in smaller series or single cases specifically of Landau-Kleffner syndrome and in a subset of children with DEE-SWAS treated with combined KD and corticosteroid therapy (63, 123). Cognitive outcomes across this literature are similarly inconsistent: full-scale IQ failed to improve, or worsened, in the majority of children in one cohort despite seizure control (124), while others have reported IQ gains exceeding 10 points and improvements in language, behavior, gait, and visual agnosia, or IQ normalization, in a subset of patients (125); the most robust and consistent cognitive and language recovery has again been reported specifically in Landau-Kleffner syndrome (123).

In a systematic review of six ESES studies involving 38 patients, >50% seizure reduction occurred in 13/32 (41%), EEG improvement in 18/34 (53%), complete EEG normalization in 3/34 (9%), and neurocognitive improvement in 17/38 (45%), with better outcomes reported in smaller early series than in larger, later cohorts (123, 126, 127). In a single-center study, combining KD with oral corticosteroids allowed 8 of 13 steroid-dependent patients to discontinue steroid therapy (63). KDT may also be particularly effective in selected genetic disorders, including Koolen–de Vries syndrome; however, it is not generally considered an early-line treatment (128).

### 2.8 Hemiconvulsion-hemiplegia-epilepsy syndrome

Hemiconvulsion-hemiplegia-epilepsy (HHE) syndrome typically affects children under 4 years of age, following prolonged unilateral (hemiclonic) febrile status epilepticus, with subsequent hemiplegia and, in many cases, chronic drug-resistant focal epilepsy. Evidence supporting KDT in HHE syndrome is limited primarily to case reports. In one small series of three children treated with KD for childhood refractory status epilepticus, a 3-year-old girl with HHE syndrome achieved seizure cessation on day 7, coinciding with the development of ketosis after the diet had been initiated on day 6 of illness (129). Another report described a 17-month-old girl with Phelan–McDermid syndrome due to a terminal 22q13 deletion involving *SHANK3*, in whom seizure control was achieved with a multimodal treatment strategy that included high-dose ketamine and initiation of KD (130).

### 2.9 Febrile infection-related epilepsy syndrome (FIRES) and new-onset refractory status epilepticus (NORSE)

Unlike most DEEs, in which KD is primarily employed to reduce the burden of chronic, recurrent seizures, FIRES/NORSE presents a fundamentally different clinical challenge: the diet is deployed as an acute, emergency intervention to terminate super-refractory status epilepticus (SRSE) in a previously healthy child (131). Consequently, the therapeutic goals and outcome measures used to judge efficacy in FIRES differ substantially from those used in other DEEs; rather than tracking long-term seizure-frequency reduction as the primary endpoint, studies in FIRES emphasize time to seizure/status-epilepticus resolution, time to ketosis, ability to wean anesthetic infusions, ICU and hospital length of stay, and functional/neurological outcome scores such as the modified Rankin Scale (mRS), the Pediatric Cerebral Performance Category Scale (PCPCS), and the Glasgow Outcome Scale (GOS) (132, 133).

The biological rationale for KD use in this setting centers on its anti-inflammatory and immunomodulatory properties rather than solely on its anticonvulsant mechanisms (134, 135). Early reports first suggested therapeutic potential: an initial retrospective series found KD efficacious in only one of four children, but a subsequent cohort from the same institution reported a markedly higher response, with six of seven patients responding favorably (136, 137). In a 15-year single-center experience, 31 children with SRSE (21 with NORSE, 14 of whom had FIRES) were started on KD, and electroclinical resolution of SRSE occurred in 54.8% of patients; at last follow-up, 7 patients were seizure-free and 11 had favorable functional outcomes (132). A separate single-center cohort of 27 children found that the KD group had significantly lower mortality, lower convulsion frequency at follow-up, reduced ASM burden, and higher GOS scores compared with those not treated with KD (133). Comparative studies have also examined KD against other targeted immunomodulatory therapies: a retrospective cohort comparing tocilizumab, KD, and first-line immunotherapy in 36 children found that while tocilizumab achieved more rapid seizure control within the first week, the KD group demonstrated superior EEG improvement by three months, suggesting a delayed but sustained therapeutic benefit (138). Based on this rationale, international consensus guidelines now recommend continuing KD for a minimum of three months into the post-acute phase in patients who have responded during the acute setting (131). A separate study evaluating vagus nerve stimulation combined with KD vs. KD alone found significantly better functional outcomes by modified Rankin Scale in the combination group, although no significant difference emerged in electrographic seizure duration between groups (139). In a larger multicenter cohort of 41 children, however, no significant difference in outcome (measured by the Clinical Assessment Scale in Autoimmune Encephalitis) was found between those who received KD and those who did not (140), and real-world surveillance data indicate that KD use in NORSE management has nonetheless increased over time despite this heterogeneity in outcome data (141). Cognitive outcomes, however, have remained a concern even among diet responders—two children treated with a 4:1-ratio KD during the acute and chronic phases of their illness achieved seizure control but were left with moderate cognitive impairment (IQ: 71 and 62) despite otherwise successful seizure management (142).

Rapid achievement of ketosis is particularly important in FIRES/NORSE, where treatment is time-sensitive, and available data indicate that early ketosis is feasible. In one series, 13 of 16 children reached ketosis within two days of diet initiation (143), while a comparative study of induction routes found that parenteral ketosis induction achieved target ketosis faster than enteral induction, without a difference in ultimate efficacy or time to SRSE termination (144). Taken together, these data support KD as a biologically plausible and increasingly utilized adjunct in the acute management of FIRES/NORSE.

## 3 Gene-specific and etiology-defined epilepsy syndromes

Advances in clinical exome and genome sequencing over the past decade have transformed a large fraction of previously “unknown etiology” DEEs into monogenic diagnoses, and this shift has direct implications for KDT. At one end of the spectrum are a small number of genetic-metabolic DEEs in which KD can serve as first-line therapy; GLUT1 deficiency syndrome due to *SLC2A1* variants (in which ketone bodies cross the blood–brain barrier independently of the GLUT1 transporter and serve as an alternative cerebral fuel) and pyruvate dehydrogenase complex (PDC) deficiency (in which fatty-acid and ketone-body oxidation generate acetyl-CoA through pathways that bypass the PDC-dependent conversion of pyruvate to acetyl-CoA) are the clearest examples (145). One study in GLUT1 deficiency reported that 67% of patients (41 of 61) became seizure-free, with 68% of those patients achieving freedom from seizures in less than a week and 76% within a month (146). Additionally, another study reported that several aspects of neuropsychological function in GLUT1 deficiency improved after 6–17 months of dietary treatment (147). In silico genomic and proteomic analyses of GLUT1 deficiency patients with an atypical lack of response to KDT may provide further insight into the biological mechanisms underlying resistance to KDT (148). Similar to GLUT1 deficiency syndrome, KD had a beneficial effect on epilepsy in all 19 patients with PDC deficiency in one study; in half, seizures disappeared within 1 year (149). EEG improved in 4/5, ataxia in 6/6, and at least minimal neurocognitive improvement occurred in 11/12. Another study in PDC deficiency reported that early diet initiation was associated with increased survival and better cognitive development (150).

At the other end of the spectrum, gene-specific studies have identified a growing number of genetic DEEs in which KD is used as a nonspecific, broad-acting antiseizure therapy, generally after failure of two or more ASMs, supported mainly by case series and small cohorts. Ko et al. conducted one of the first systematic attempts to summarize KD efficacy according to underlying genetic etiology in DEE and found that ≥90% seizure reduction was reported in 5/6 patients with *KCNQ2* variants, 4/4 with *STXBP1* variants, and 3/3 with *SCN2A* variants, whereas none of 10 patients with *CDKL5* variants met this stringent response threshold (151). A meta-analysis reported gene-specific responder rates (an overall rate of 58% among 849 patients) of 96% for *SLC2A1* (*n* = 87), 73% for *SCN2A* (*n* = 16), 64% for *SCN1A* (*n* = 277), 60% for *POLG* (*n* = 5), 54% for *STXBP1* (*n* = 27), 42% for *KCNQ2* (*n* = 9), 36% for *PCDH19* (*n* = 8), 24% for *KCNT1* (*n* = 70), 23% for *CDKL5* (*n* = 111), and 15% for *SCN8A* (*n* = 5) (152). The corresponding gene-specific seizure-freedom rates (an overall rate of 29%) were 67% for *SLC2A1* (*n* = 80), 26% for *STXBP1* (*n* = 15), 25% for *SCN2A* (*n* = 13), 10% for *SCN1A* (*n* = 209), 2% for *KCNT1* (*n* = 48), and 0% for *CDKL5* (*n* = 40).

In another cohort, identification of a pathogenic or likely pathogenic variant was associated with a more favorable response to KDT (153). At 2 years, 17.3% of patients were seizure-free, while an additional 25% achieved greater than 90% seizure reduction (153). Particularly favorable responses were observed in individuals with disease-causing variants in *SLC2A1*, *SLC6A1*, *SCN1A*, *STXBP1*, and *PAFAH1B1* (154, 155). Clinically meaningful benefit was also reported with variants in *DEPDC5*, *GLDC*, *KCNT1*, *PDHA1*, *SLC25A12*, and *TSC1* (156, 157). In contrast, variants in *COL4A1* and *DYNC1H1* were among those associated with limited or absent benefit. Additional gene-specific observations suggested favorable KDT responses in patients with pathogenic variants in *CSNK2A1*, *FARS2*, *GABRB3*, *GRIN1*, *KCNA2*, *KCTD3*, *STX1B*, and *SLC16A2*. Conversely, poor response was reported in association with variants in *CLN5*, *GLI3*, *MACF1*, *MAGEL2*, *NANS*, *IKBKG* (NEMO), *RORB*, *SLC17A5*, and *UFSP2*. When genes were categorized according to biological function, transporter-related disorders (*SLC2A1*, *SLC12A5*, *SLC25A12*, *SLC16A2*, *SLC17A5)* showed the most favorable outcomes. Beyond *SLC2A1*, several independent studies of *SLC25A12*-related disease have reported marked responses to KDT, including improvements in seizure control, cognition, motor function, and brain myelination (158, 159). Disorders involving other membrane-associated proteins, including ion channels and neurotransmitter receptors, also demonstrated relatively good efficacy. By comparison, genes involved in cellular structural integrity or homeostatic regulation (*COL4A1, UFSP2, MAGEL2, PIGT)* were associated with the least favorable dietary response (153).

Several other genetic DEEs have been the subject of small case series suggesting a potentially favorable response to KD. In tuberous sclerosis complex, the largest dedicated cohort included 31 patients, of whom 67.7% achieved ≥50% seizure reduction at 3 months and 32.3% maintained this response at 24 months, including 19.4% who were seizure-free (160). Among 104 individuals with *CDKL5* deficiency disorder exposed to KDT, caregiver-reported improvement occurred in 88% of those with available outcome data, although only 32% remained on treatment and loss of efficacy was the most common reason for discontinuation, whereas a subsequent meta-analysis of 193 patients estimated a substantially lower responder rate of 18%, with some degree of benefit reported in up to 50% (161, 162). In an Angelman syndrome-specific series, 23 patients treated with LGIT showed favorable seizure outcomes, with 22% becoming seizure-free, 43% experiencing seizures only under specific circumstances such as illness, and 30% having a substantial reduction in seizure frequency (163). Several additional studies in Angelman syndrome have also reported favorable efficacy (164, 165). Conversely, data in Rett syndrome remain sparse, although a recent nine-patient series reported ≥50% seizure reduction in 67% at 6 months and 44% at 12 months (166).

## 4 Adverse effects of ketogenic diet therapy and monitoring requirements

Adverse effects are common across all DEE syndromes reviewed here and represent the principal driver of diet discontinuation over time, as reflected in the sharp attrition in treatment retention reported across nearly every cohort discussed above (167) (Table 5). Given the adverse-effect profile summarized above, structured clinical and biochemical monitoring is essential to the safe and sustained use of KDT (Table 6).

**Table 5:** Adverse effects of KDT in DEEs.

Category	Adverse effects	Notes
Gastrointestinal	Constipation, vomiting, diarrhea, abdominal pain, gastroesophageal reflux	Most common adverse-effect category across nearly all reviewed cohorts; frequently cited reason for dose adjustment or diet liberalization
Hepatic/Pancreatic	Pancreatitis (rare, can be fatal), protein-losing enteropathy/hypoproteinemia, cholelithiasis, hepatitis	Enteropathy often reflects an underlying pathology (reported in an *STXBP1*-related DEE) and may respond to reducing the fat:carbohydrate ratio rather than full discontinuation
Hematologic	Neutropenia, decreased platelet aggregation/bruising, increased hemoglobin/hematocrit	Neutropenia associated with higher urinary ketones and longer diet duration without increased infection risk; desmopressin may improve platelet-related bruising
Metabolic/biochemical	Hypoglycemia (especially during induction), dyslipidemia (elevated LDL cholesterol and triglycerides), metabolic acidosis, symptomatic hyperketosis	Requires baseline and periodic lipid panel and metabolic monitoring; parenteral induction achieves ketosis faster and requires close biochemical monitoring during transition
Renal	Nephrolithiasis (kidney stones), renal tubular acidosis	Risk increased with concurrent carbonic anhydrase inhibitor use (topiramate, zonisamide, acetazolamide); adequate hydration and urine alkalinization/citrate supplementation may reduce risk
Growth and nutritional	Growth deceleration/linear growth impairment, weight loss or poor weight gain, micronutrient deficiencies (selenium, zinc, vitamin D, carnitine)	Requires dietitian-supervised vitamin/mineral supplementation and periodic growth monitoring, particularly relevant given prolonged diet duration in some DEEs
Bone health	Reduced bone mineral density, increased fracture risk	Particular concern in syndromes with baseline immobility or reduced weight-bearing
Cardiac	QT prolongation, cardiomyopathy (rare)	Baseline and periodic ECG/echocardiogram monitoring recommended, particularly with prolonged diet duration or in metabolic/mitochondrial disease
Micronutrient-related	Selenium-deficiency-associated cardiomyopathy, carnitine deficiency	Reinforces need for supplementation and periodic laboratory monitoring
Energy/behavioral	Lethargy, irritability during induction (“keto flu”), reduced alertness in some patients	Often transient; may improve with slower induction or ratio adjustment
Dermatological	Prurigo pigmentosa (“keto rash”)	Responds to carbohydrate liberalization or tetracyclines; no clear population-specific predilection
Neurological (rare)	Basal ganglia injury with chorea/ataxia	Anecdotal reported case with reversible MRI/MRS changes but persistent movement disorder; possible link to underlying mitochondrial vulnerability

**Table 6:** Recommended monitoring for children on KDT.

Monitoring domain	Before KDT initiation	During maintenance
Clinical and nutritional assessment	Height/length, weight, BMI or weight-for-length, growth trajectory, dietary intake, feeding route, fluid intake, and food allergies/intolerances	Growth, nutritional adequacy, adherence, fluid intake, supplements, gastrointestinal tolerance, seizure response, and adverse effects at each visit
CBC and metabolic profile	CBC with platelets; electrolytes including bicarbonate, calcium, glucose, total protein, BUN/urea, creatinine, albumin, and liver enzymes	Repeat at routine follow-up, generally every 3 months during the first year and every 6 months thereafter
Fasting lipid profile	Total cholesterol, LDL-C, HDL-C, and triglycerides	Repeat at approximately 3 months and periodically thereafter
Vitamin and mineral assessment	25-hydroxyvitamin D; consider magnesium, phosphorus, zinc, copper, and selenium when clinically indicated	Periodic vitamin D assessment; additional micronutrients according to diet duration, nutritional status, growth, and symptoms
Carnitine and metabolic screening	Acylcarnitine profile; consider free and total carnitine, plasma amino acids, and urine organic acids, particularly in young children or when the etiology is unclear	Free and total carnitine periodically, especially with valproate, poor nutrition, fatigue, hepatic dysfunction, or difficulty maintaining ketosis
Urine and renal monitoring	Urinalysis; consider urine calcium-to-creatinine ratio in patients at increased stone risk	Periodic urinalysis; urine calcium-to-creatinine ratio and renal ultrasonography if hypercalciuria, hematuria, pain, or nephrolithiasis risk is present
Ketosis monitoring	Baseline ketones optional	Blood BHB or urine ketones during initiation and dietary adjustment, and thereafter when adherence or efficacy is uncertain
ASM monitoring	Review all ASMs and carbohydrate-containing formulations; obtain levels when clinically relevant	ASM levels when toxicity, altered seizure control, adherence concerns, or drug–diet interactions are suspected
Bone and cardiac assessment	DEXA and cardiac testing are not routinely required; consider with pre-existing risk factors	Consider DEXA after prolonged treatment or earlier with fractures, immobility, poor growth, or other bone-risk factors; ECG or echocardiography as clinically indicated

ASM, antiseizure medication; BHB, β-hydroxybutyrate; BMI, body mass index; BUN, blood urea nitrogen; CBC, complete blood count; DEXA, dual-energy x-ray absorptiometry; ECG, electrocardiogram; HDL-C, high-density lipoprotein cholesterol; KDT, ketogenic dietary therapy; LDL-C, low-density lipoprotein cholesterol.

Routine follow-up is generally performed every 3 months during the first year and every 6 months thereafter, with closer monitoring in infants and patients at increased nutritional or metabolic risk.

## 5 Future directions

Several gaps in the current evidence base warrant prioritization in future research. First, comparative-effectiveness data for KDT vs. other antiseizure therapies, including neuromodulation, remain limited outside IESS and, to a lesser extent, FIRES, precluding evidence-based treatment sequencing and necessitating continued reliance on retrospective cohort comparisons.

Second, reporting of nonseizure outcomes across the KDT literature remains inconsistent, particularly for neurodevelopment, quality of life, and EEG changes. Future KDT trials and registries should prospectively adopt standardized endpoints to facilitate meaningful cross-study comparisons and, ultimately, individual patient data meta-analyses.

Third, predictors of KDT response require evaluation in larger cohorts. Although emerging data suggest that response may vary by genetic etiology, both gene-specific and DEE phenotype–specific studies are needed because the same genetic cause can manifest across distinct electroclinical phenotypes. Integration of multi-omics approaches, including genomics, transcriptomics, proteomics, and metabolomics, may further help identify biological signatures and pathways associated with KDT response and enable more precise patient selection (168).

Fourth, tolerability remains the single most consistent limiting factor identified across syndromes, with adherence declining sharply over the first 6–12 months. Comparative studies of KD against more liberalized formulations suggest broadly comparable efficacy with improved tolerability for the less restrictive regimens in several syndromes, and further trials directly comparing formulations within specific DEE syndromes would help refine which patients are best served by the classic 4:1 ratio vs. a more liberalized approach. Further research should evaluate potentially better-tolerated formulations, including MCT- and C10-enriched diets, ketone esters, ketone salts, and triheptanoin.

Fifth, KDT has often been initiated relatively late in infantile-onset DEEs; however, one study suggested that initiation before 9–18 months of age may be associated with higher seizure-freedom rates at 3, 6, and 12 months (36). Although breastfeeding may complicate implementation, human milk can be continued during KDT using measured expressed breast milk or structured, time-limited breastfeeding without complete weaning. The potential effects of early KDT on infant growth remain an important concern and require further study.

Finally, the cost, availability, and resource intensity of dietary therapies raise important health-equity concerns. Successful implementation generally requires a multidisciplinary team that may include a dietitian, neurologist, geneticist, advanced practice provider, nurse, and social worker. Treatment also involves careful macronutrient calculation, precise ingredient measurement, routine vitamin and mineral supplementation, and regular laboratory monitoring to maintain nutritional adequacy and identify metabolic complications. Medications may need to be converted to low-carbohydrate formulations or compounded, further increasing cost. Restrictive dietary requirements can disrupt family routines, limit social participation, and increase food-related expenses, while insurance coverage for specialized formulas, foods, and supplements varies substantially by geography and payer. Feasibility may also depend on caregiver health literacy, cultural and religious practices, food allergies, and dietary preferences. Future efforts should prioritize simplified protocols, culturally adaptable resources, telehealth-supported dietetic care, and more consistent insurance coverage to reduce financial and geographic disparities in access.

## 6 Conclusion

KD and its liberalized variants have an increasingly well-supported role across the spectrum of DEEs, encompassing classic electroclinical syndromes, a growing number of gene-specific disorders, and acute, life-threatening presentations such as super-refractory status epilepticus in FIRES and NORSE. Across this heterogeneous literature, efficacy cannot be captured by a single outcome measure, because benefits may manifest as electroclinical remission of spasms, freedom from drop attacks, preservation or recovery of language, termination of status epilepticus, or stabilization of an underlying metabolic disorder. A syndrome-specific, endpoint-sensitive approach to clinical decision-making and future trial design will therefore be essential to fully define the therapeutic potential of dietary therapy in DEEs. At the same time, sustained clinical benefit will depend on addressing adverse effects, substantial long-term attrition, treatment burden, and persistent health-equity barriers.
